# Antimicrobial resistance burden estimates from the bottom-up: research priorities for estimating the impact of antimicrobial resistance in Brazil

**DOI:** 10.1016/j.ijregi.2024.100558

**Published:** 2025-01-04

**Authors:** Katherine Keenan, Carlos Roberto Veiga Kiffer, Érico V.S. Carmo, Juliana Silva Corrêa, André Luiz de Abreu, Adriano Massuda, Ana Christina Gales, Arnaldo Lopes Colombo, the Institute of Antimicrobial Resistance of São Paulo (ARIES) group

**Affiliations:** 1 School of Geography and Sustainable Development, University of St Andrews, St Andrews, United Kingdom; 2 Escola de Administração de Empresas da Fundação Getúlio Vargas, São Paulo, Brazil; 3 Escola Paulista de Medicina – Universidade Federal de São Paulo (UNIFESP), São Paulo, Brazil; 4 Secretaria de Vigilância em Saúde, Ministério da Saúde (SVS/MS), Brasília-DF, Brazil

**Keywords:** Antimicrobial resistance, Brazil, Clinical burden, Study design, Policy

## Abstract

Recent estimates of deaths attributable to bacterial antimicrobial resistance (AMR) highlight the immense public health threat of AMR to healthcare systems, economies, and communities in Latin America. Although global modelling studies generate important statistics to motivate and guide global and national agendas, their complex methodology and aggregation mean that they have a more limited impact at the local scales where AMR is experienced and tackled. At the same time, it is increasingly recognised that we need to study and design AMR policies ‘from the bottom-up’, drawing on data and perspectives that ensure local ownership of the research and policy agenda. But how do we integrate ‘bottom-up’ perspectives into AMR burden estimation? Brazil is used as a case study to illustrate the importance of this approach. Brazil's vast and decentralised healthcare system would benefit from robust regional estimates of AMR's clinical, economic, and social burdens to move political decision-making and design appropriate interventions. We report on recommendations gathered from interdisciplinary stakeholder exercises and propose strategic priorities for estimating the AMR burden in Brazil at subnational scales of governance. These include focusing on individual-level data linkages at various scales; capturing public and private healthcare systems; understanding AMR inequalities; and capturing linked clinical, economic, and social burdens.

## Highlights

• We need to develop antimicrobial resistance (AMR) burden estimates at policy-relevant scales.
• We use Brazil to illustrate the importance of ‘bottom-up’ approaches for estimating AMR burdens.
• Brazilian stakeholders prioritise subnational AMR studies covering public/private sectors.
• In the Brazilian context, understanding social inequalities in AMR outcomes is the key.

## Introduction

In the Americas, in 2021, the Global Research on Antimicrobial Resistance (GRAM) team estimated that 78,000 deaths (67,700-88,600) were directly attributable to bacterial antimicrobial resistance (AMR) [1], more than breast cancer for the same period [2]. These headline statistics are vital for making AMR visible in a crowded global health policy landscape. Crucially, they also provide estimates that are comparable across time and space, enable the tracking of disease trends at global, regional, and national levels, while also highlighting disparities and providing useful benchmarks for measuring progress. However, behind the estimates is a highly complex methodological exercise, often seen as a ‘black box’ by policymakers. This is partly due to the nature of AMR which is not a well-defined disease but has long been a hidden constituent of many diverse health problems, including bacterial infections acquired in both hospital and community settings, such as sepsis and tuberculosis. The data available for the identification of resistant cases are diverse, and many issues exist, including data quality, representativeness, digitisation, and accessibility, which are worse in low- and middle-income countries (LMICs). There is a shortage of studies linking patients to pathogens and their outcomes, especially in LMICs; therefore, the methodology is highly inferential. There is often a lack of standardisation in data collection methods, laboratory procedures, and reporting formats, making it challenging to compare and interpret AMR data from different sources. Therefore, the estimates developed by the GRAM team aggregate disparate data across various scales and combine these to create numbers at national or supranational scales that have high degrees of uncertainty. This process creates a sense of distance between the numbers produced and the spaces in which AMR is experienced, a gap which blurs local perspectives.

Meanwhile, it is increasingly argued that AMR agendas should be driven from the ‘bottom-up’. AMR is experienced and managed at smaller scales, and the drivers and realities are dramatically different in the Global South than in the Global North [3]. Local ownership of AMR policy and research agenda is also crucial if National Action Plans are to effect change [4]. We are encouraged to work with a people-centred approach to AMR [5], and we need to build forms of evidence that capture and represent the perspectives of people rather than pathogens, as well as prioritise connections with on-the-ground realities and priorities. How can we reconcile the thirst for both global and local evidence of the AMR burden, and how can we use these to drive action at the scales that matter? We illustrate the challenges and opportunities of estimating the burden ‘from the bottom-up’ using the case study of Brazil, a middle-income country with multiple decentralised layers of governance and AMR decision-making.

## AMR clinical burden estimation: integrating bottom-up and top-down perspectives

What would it mean to measure AMR burden from the ‘bottom-up’? An ideal example of a bottom-up study would be an observational cohort study recommended by the World Health Organization Global Antimicrobial Resistance and Use Surveillance System (WHO GLASS), which focuses on a certain type of infection [6]. The protocol emphasises the local context during the design phase, including the need to consider what is reliably measured, sampling biases, and appropriate comparator cohorts. A growing number of such studies have been conducted on bloodstream infections [7,8]. Cohort studies are more flexible in responding to the local context of their design, considering what matters to the people and structures in particular places. They also have the ability to collect a variety of prospective data to capture social and economic burdens, as well as clinical data. However they are expensive and time-consuming. Well-designed cross-sectional epidemiologic studies on specific target populations [9] are useful for providing auxiliary estimates. Both methods can be scaled up to take a ‘middle-range’ approach which leverages electronic patient records to link patient outcomes and pathogen data at larger scales [10,11], providing lower-cost ways of conducting cross-sectional and cohort studies that generate burden estimates, while still not losing touch with the context from which they are drawn.

**Figure 1:**
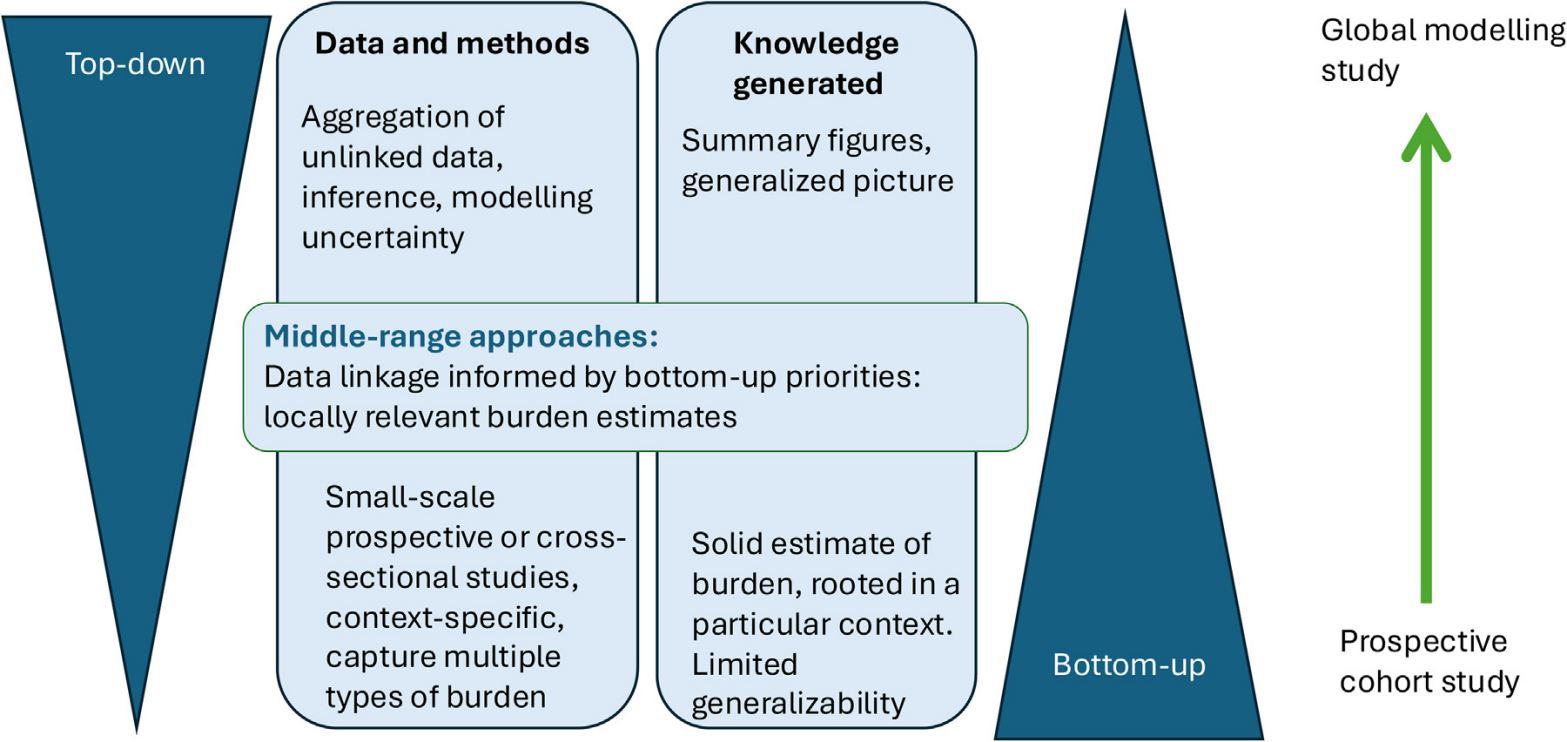
Integrating data at different scales to measure antimicrobial resistance burden.

At the other end of the scale, what does a 'top-down' approach to estimating the AMR burden look like? The methodology used by the GRAM team illustrates how to integrate global evidence collected at various scales in the absence of a direct linkage between people, pathogens, and outcomes. Evolving from the earlier O'Neill reviews in 2014-16 [12,13] and regional efforts from Europe (European Centre for Disease Control and Prevention [14]) and the United States (U.S. Centers for Disease Control and Prevention [15]), they combined data measuring components including infection prevalence, cause of death, surveillance data on AMR, and literature from smaller-scale cohort studies on case-fatality. The latest modelling exercise draws on 520 million individual records [1]. The inferential methodology can be considered ‘top-down’ in its logic. It starts by identifying all bacterial sepsis deaths, estimates typical levels of resistance in many drug-bug combinations responsible, and then combines these to narrow down further to estimate the fraction of bacterial sepsis deaths which are both associated with and directly attributable to bacterial AMR. In doing so, it incorporates data from a multitude of ‘bottom-up’ perspectives, including smaller-scale cohort studies which do provide linkage between people-pathogen and outcome. The contrast between bottom-up cohort studies and top-down modelling (Figure 1) is not just methodological; it is also one of the perspectives and intended audiences. By their nature, cohort studies are rooted in context, telling the story of one site (geographical or clinical). In contrast, global burden studies are designed to generate a big picture to guide health agendas at larger scales.

## Priorities for estimating AMR burden in Brazil

Brazil is the world's fifth-largest nation, making the Brazilian Public Health System (Sistema Único de Saúde, or SUS) one of the largest health systems globally. The country is governed in a decentralised manner, comprising a federal system of 27 states and more than 5500 elected municipal governments. As a universal healthcare system, the SUS is responsible for providing access to health for all 203 million Brazilian citizens. Of this total, around a quarter had access to the private healthcare system. The federal government body, through the Ministry of Health (MoH), is responsible for the coordination and supervision of the SUS and, therefore, for developing, proposing, and helping to implement national health policies with states and municipalities. The MoH can influence policy agendas at regional levels; however, states and municipalities often have different priorities, unequal resources (such as health system structures and capacities), and autonomy, giving them the right to decide whether and how to engage with some of the priorities set by the MoH in the national health policy agenda. In terms of AMR initiatives, a recent study involving federal-level stakeholders in Brazil [16] highlights that AMR is still not considered a policy priority. Although the National Action Plan (BR-NAP) was strongly influenced by the global health policy agenda, at the national level, there is still a lack of visibility and political will. There is a need to engage state and municipal governments to build evidence and awareness of the clinical, social, and economic impacts of AMR. It is worth noting that while Brazil has a national plan to combat AMR, it lacks a dedicated National Program for AMR, similar to those in place for HIV/hepatitis, tuberculosis, immunisation, and other health priorities. The absence of such a program means that planned activities may not necessarily be executed because of the lack of specific budget allocation for AMR initiatives.

Efforts have been made to estimate the burden of infection and resistance. Table 1 presents a summary of (mainly national-level) evidence from studies of common community- and hospital-associated infections with their respective resistant pathogens. Some studies have drawn from national data of the Brazilian Health Regulatory Agency (ANVISA) [[21], [22], [23], [24], [25], [26]], while others have used hospital data with more limited geographical scope [19]. There is substantially more information on the prevalence of healthcare-associated infections, especially those in intensive care units (ICUs) [26], compared on community-acquired infections. For example, an initiative has been undertaken to identify resistance patterns at the nationwide level, disaggregated by geographical macroregions and year, but has focused only on multidrug-resistant pathogens from ICUs [26]. Studies have also tended to describe prevalence and not link it to clinical outcomes. Thus, these indicate areas for future burden studies.

**Table 1:** Overview of the most common community- and hospital-associated infections with their respective pathogens and resistance levels in Brazil.

**Infection**	**Point prevalence (% range)**	**Pathogen**	**Resistance of interest**	**Point prevalence (% range)**
**Pneumonia**				
Community setting	NA	*S. pneumoniae*	Reduced susceptibility to ceftriaxone	20.6 [17]
Health-care setting	3.6 (2.7-4.4) [18]	Enterobacterales	Carbapenem	12.5-44 [18,19]
**Urinary tract infection**				
Community setting	NA	*E. coli*	Reduced susceptibility to ciprofloxacin	14.3-35 [20,21]
Health-care setting	1.4 (0.8-1.8) [18]	*E. coli*	Extended spectrum beta-lactamases	11-51 [21,22]
**Bloodstream infection**				
Health-care setting	2.9 (1.5-3.3) [18]	Enterobacterales	Carbapenem	19-26.9 [22,23]
		*S. aureus*	Methicillin-resistant *S. aureus*	58-60.7 [22,24]
		*P. aeruginosa*	Carbapenem	24-40.6 [22,25]
**Surgical site infection**				
Health-care origin	1.5 (0.9-3.1) [18]	*S. aureus*	Methicillin-resistant *S. aureus*	58-60.7 [22,24]

The GRAM study team estimated that 33,329 deaths were directly attributable to bacterial AMR in 2019 [27], concentrated in neonates and those aged >75 years. In line with other countries in the Americas, the main pathogens responsible were *Staphylococcus aureus, Escherichia coli*, and *Klebsiella pneumonia*. Methicillin-resistant *S. aureus* was the deadliest drug-bug combination, followed by carbapenem-resistant *K. pneumonia and Pseudomonas aeruginosa*. Although individual-level data linking patient outcomes to pathogens were not used, the calculations drew on resistance data from national surveillance systems (for example, BR-GLASS and Latin American Network for Antimicrobial Resistance Surveillance), multiple causes of death data, hospital discharge summaries, and pre-existing literature across several of the most populous states. However, some Brazilian policy stakeholders believe that these aggregated AMR burden estimates are not convincing enough to drive action at national and subnational levels [16]. The mortality figures presented in these studies are relatively low compared to other Brazilian health threats, particularly in the context of technical and resource limitations. There is a disconnect between global studies estimating AMR burden - often published in English in international journals - and the practical needs of health managers at state and municipal levels. Addressing the policy-science gap at the national level is essential for fostering ownership of the agenda. Furthermore, global burden estimates often frame AMR as a 'future threat' which, combined with the factors above, has contributed to its perception as an 'imported agenda', which does not align with local competing priorities.

Therefore, evidence linking infections to resistance and outcomes at smaller scales is required. However, bottom-up cohort studies are scarce and limited. A recent systematic review [28] identified 29 cohort studies that estimated attributable mortality due to bacterial AMR in Brazil, published from 2001-2020. Most retrospective cohort designs are based on admission to hospital ICUs, the picture of community burden and nonbacterial infections is very limited. One hope is for better data sharing and linkages to provide better regional estimates. A recently established platform for clinical data sharing, IMPACTO-MR [29] links patient outcomes and sociodemographic, clinical, and health data across public- and private-sector facilities in many Brazilian states to conduct observational and intervention studies on patients in intensive care settings. Although still in its infancy, such an approach has the potential to improve understanding of the clinical and economic burden of AMR in hospital settings in the future and to be responsive to emerging threats. Another initiative is the pilot of the national antimicrobial surveillance program, BR-GLASS [30], established by the MoH, which aims to understand the impact of AMR in the country and support the decision-making process at the national level. The rich data collected through the SUS across many locations and managed by the Department of Informatics of the SUS (DATASUS) has great potential for integration and analysis. Additional sources can augment this, including commercial data on antimicrobial consumption [31], private-sector hospital data [32], and outpatient networks. However, significant gaps remain in patient data capture and linkage services to individual outcomes beyond mortality.

## A research agenda for holistically measuring AMR burden from the ‘bottom-up’

In response to this challenge, in late 2023, the newly established Centre for Research, Innovation, and Dissemination (CEPID) funded by The State of São Paulo Research Foundation (FAPESP), the Antimicrobial Resistance Institute of São Paulo (ARIES), developed a series of workshops between experts and stakeholders to discuss priorities for estimating the interlinked clinical, social, and economic burdens of AMR. Across the three hybrid events, which together were attended by over 100 participants, the following gaps, challenges and priorities were identified:

• **Focus on relevant levels of governance**: the multiple layers of health governance in Brazil mean that refining burden estimates at the state and municipal levels is important for driving policy will, social engagement, actions, and solutions.
• **Data linkage**: data gaps are vast and challenging; however, priority should be placed on linking patient data, outcomes, and pathogen resistance data at the individual level and tracking them longitudinally.
• **Health inequalities:** the question of equity is particularly critical in Brazil, where residents experience vast differences in their vulnerability to infection and access to care [33]. Parallel private and public medical systems create different journeys for patients with differential access to diagnostics, treatment, and follow-up [33]. Similarly, vulnerability to infection is likely to be affected by socioeconomic, geographic, and racial disparities, similar to other transmissible conditions [34,35]. Therefore, linkages should include data from public and private sectors which measure and match demographic and socioeconomic factors.
• **Clinical, economic and social burdens**: efforts to track AMR burden have often focused on clinical aspects and missed opportunities to understand linkages with economic and social impacts.

Taking these priorities together, the ARIES group agreed that, although top-down inferential approaches are useful for highlighting the large scale of the problem to drive solutions and change, there is a need to focus on burden estimates driven by local priorities at smaller geographical scales. This led the group to propose developing a range of longitudinally linked cohort studies in a well-defined areas for specific conditions, incorporating measurements of clinical, economic, and social burdens using interdisciplinary forms of data collection. This detailed picture can be combined with pre-existing medical and environmental data using data science techniques. Such an approach, which it is co-designed with local policymakers, clinicians and researchers, would produce more impactful burden estimates from the ‘bottom-up’, and could be used to develop and study interventions.

These detailed ground-up perspectives would provide insights into a middle-range data observatory that collates data at the local, regional, and national scales. This would draw on the rich data collected through the SUS at its many locations managed by the Department of Informatics from the SUS (DATASUS), which has great potential for integration and analysis. Additional sources can augment this, including commercial data on antimicrobial consumption [31], private-sector hospital data [32], and outpatient networks.

The ARIES group also identified the need for new translational methods and tools to translate academic AMR research to policymakers. This requires significant advocacy and consideration from academia, particularly when transforming complex models and data into locally and regionally acceptable information. In Brazil, the MoH has a long tradition of producing data and monitoring the performance of many diverse conditions (e.g., National Immunisation Program, HIV, Tuberculosis, etc.), but it still lacks adequate methods for producing information and monitoring such a transversal and complex field as AMR. Thus, initiatives to estimate the AMR burden from the bottom-up must be communicated using well-established information methods, such as epidemiological bulletins, in close conversations with the MoH and state secretariates, to ensure successful AMR strategies.

Such an integration of ‘middle-range’ and ‘bottom-up’ approaches is also relevant for many other Latin American nations and LMIC contexts. AMR policies and data must be generated at the community level if they have a local impact. AMR priorities and policies have long been imported from the Global North which has undermined ownership and buy-in. Estimating AMR burden from a ‘bottom-up’ serves several functions: it can help build awareness at scales that matter, stimulate political will, and sustainably engage various stakeholders at different regional and local levels, including health professionals, patients, and society. Ultimately, a bottom-up approach can help us to bridge the research-policy-society divide on AMR.

## Declarations of competing interest

The authors have no competing interests to declare.
